# The Roles of Transmembrane Domain Helix-III during Rhodopsin Photoactivation

**DOI:** 10.1371/journal.pone.0017398

**Published:** 2011-02-25

**Authors:** Wen-bin Ou, Tingfang Yi, Jong-Myoung Kim, H. Gobind Khorana

**Affiliations:** 1 Department of Biology, Massachusetts Institute of Technology, Cambridge, Massachusetts, United States of America; 2 Department of Biological Chemistry and Molecular Pharmacology, Harvard Medical School, Boston, Massachusetts, United States of America; University of Oldenburg, Germany

## Abstract

**Background:**

Rhodopsin, the prototypic member of G protein-coupled receptors (GPCRs), undergoes isomerization of 11-cis-retinal to all-trans-retinal upon photoactivation. Although the basic mechanism by which rhodopsin is activated is well understood, the roles of whole transmembrane (TM) helix-III during rhodopsin photoactivation in detail are not completely clear.

**Principal Findings:**

We herein use single-cysteine mutagenesis technique to investigate conformational changes in TM helices of rhodopsin upon photoactivation. Specifically, we study changes in accessibility and reactivity of cysteine residues introduced into the TM helix-III of rhodopsin. Twenty-eight single-cysteine mutants of rhodopsin (P107C-R135C) were prepared after substitution of all natural cysteine residues (C140/C167/C185/C222/C264/C316) by alanine. The cysteine mutants were expressed in COS-1 cells and rhodopsin was purified after regeneration with 11-cis-retinal. Cysteine accessibility in these mutants was monitored by reaction with 4, 4′-dithiodipyridine (4-PDS) in the dark and after illumination. Most of the mutants except for T108C, G109C, E113C, I133C, and R135C showed no reaction in the dark. Wide variation in reactivity was observed among cysteines at different positions in the sequence 108–135 after photoactivation. In particular, cysteines at position 115, 119, 121, 129, 131, 132, and 135, facing 11-cis-retinal, reacted with 4-PDS faster than neighboring amino acids. The different reaction rates of mutants with 4-PDS after photoactivation suggest that the amino acids in different positions in helix-III are exposed to aqueous environment to varying degrees.

**Significance:**

Accessibility data indicate that an aqueous/hydrophobic boundary in helix-III is near G109 and I133. The lack of reactivity in the dark and the accessibility of cysteine after photoactivation indicate an increase of water/4-PDS accessibility for certain cysteine-mutants at Helix-III during formation of Meta II. We conclude that photoactivation resulted in water-accessible at the chromophore-facing residues of Helix-III.

## Introduction

G-protein-coupled receptors (GPCRs), the largest known family of cell surface receptors, mediate a wide variety of signal transduction processes. Upon recognition of diverse extracellular signals including hormones, neurotransmitters, olfactants, tastants, and light, GPCRs initiate intracellular signaling by interacting with heterotrimeric G proteins [1]. Rhodopsin defines the rhodopsin-like (class A) family within the large GPCR superfamily [1] and it is the earliest GPCR for which a high-resolution crystal structure has been determined [2]. Rhodopsin consists of cytoplasmic (CP), transmembrane (TM), and extracellular (EC) domains. The mechanism by which rhodopsin is activated has been extensively characterized [2]–[14]; the most relevant studies in this regard include the recently determined crystal structure of inactive [2] and partially activate rhodopsin [7], [8]; site-directed spin labeling and double electron-electron resonance (DEER) studies [9]; the model of two protonation switches operating at the Schiff base (E113) and the cytosol (E134) [10], [11]; and the function of structural waters [12]–[14].However, the role of TM helix-III in rhodopsin photoactivation remains unclear.

Rhodopsin is composed of the 40-kDa apoprotein opsin (348 amino acids) and its chromophore 11-cis-retinal, which acts as an inverse agonist in the rhodopsin ground state [15]. It is well known that light induces rhodopsin isomerization from 11-cis-retinal to all-trans-retinal. This isomerization activates the receptor by causing movements of the TM helices, which, in turn, induce conformational changes in the CP domain that result in transition to the activated state [16]. This state is competent for binding the heterotrimeric G-protein of the rod cell transducin (Gt), and for catalysis of the uptake of guanosine triphosphate by the α-subunit of Gt, thereby initiating the enzymatic cascade that leads to light detection and ultimately to vision.

Several approaches, including electron paramagnetic resonance, nuclear magnetic resonance, and crystallization, have been used to obtain insight into the structure of rhodopsin and its conformational changes on light activation [2]–[19]. Site-directed cysteine mutagenesis followed by biochemical and biophysical analysis of rhodopsin has also extensively been employed for determining secondary and tertiary structure of CP in rhodopsin [20]–[23]. A particular sulfhydryl specific reagent, 4, 4′-dithiodipyridine (4-PDS), can be used as a probe to determine the cysteine reactivity in rhodopsin mutants. Rhodopsin carrying a free sulfhydryl group reacts with 4-PDS to form the dipyridinyl derivative. The rate of this reaction is very sensitive to the accessibility of the cysteine [16].

The aim of this study is to better understand GPCR activation by investigating the conformational changes of rhodopsin upon light activation. Cysteines introduced one at a time along the full length of the TM helices will be tested for their accessibility and consequent reactivity to sulfhydryl reagents in the dark and on light activation.

**Figure 1:**
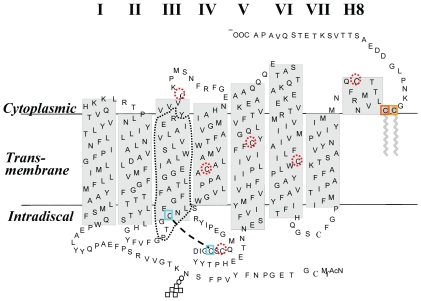
A secondary structure model of rhodopsin. Naturally occurring cysteines (dotted circles) and amino acid residues (P107-R135) mutated in this study are highlighted.

To determine the conformational changes of rhodopsin on light activation, a basal rhodopsin mutant was constructed replacing the six naturally-occurring free cysteine residues with the neutral amino acid alanine, to avoid ambiguity caused by signals derived from natural cysteines in rhodopsin. As shown in Figure 1, twenty-eight single-cysteine substituted mutants of helix-III (P107-R135) were generated based on the basal rhodopsin mutant (C140A/C167A/C185A/C222A/C264A/C316A). Single-cysteine substituted mutants on the background of the basal mutant were analyzed by rhodopsin chromophore formation, bleaching behavior, and Meta II decay. In addition, the accessibility of cysteine in each of these mutants was monitored by reaction of sulfhydryl group with 4-PDS in the dark and after illumination for 30 seconds. The results suggest that there is an aqueous/hydrophobic boundary in helix-III near G109 and I133. The chromophore-facing residues of Helix-III become water-accessible after photoactivation.

## Materials and Methods

### Materials

All-*trans*-retinal, 4-PDS and phenylmethylsulfonylfluoride (PMSF) were purchased from Sigma (St. Louis, MO). n-dodecyl-β-D-maltopyranoside (DM) was acquired from Anatrace (Maumee, OH). Antirhodopsin monoclonal antibody rho-1D4 was purified from myeloma cell lines and coupled to cyanogen bromide-activated Sepharose 4B (Sigma) as described elsewhere [24]. The nonapeptide corresponding to the C-terminal sequence of rhodopsin, which was used to elute rhodopsin samples from the antibody 1D-4 sepharose matrix, was prepared at the MIT Biopolymers Laboratory. The buffers used were the following: buffer A, PBS buffer: 137 mM NaCl, 2.7 mM KCl, 1.8 mM KH_2_PO_4_, 10 mM Na_2_HPO_4_ (pH 7.2); buffer B, buffer A plus 1% DM (w/vol); buffer C, buffer A plus 0.05% DM; buffer D, 2 mM NaPi (pH 6.0), 0.05% DM; buffer E, 2 mM NaPi (pH6.0), 0.05% DM, 100 µM nonpeptide; and buffer F, 2 mM NaPi (pH8.0), 0.05% DM.

### Construction of single-cysteine mutants P107C-R135C in vector PMT4

A synthetic opsin construct containing the mutations C140A, C167A, C185A, C222A, C264A, and C316A was generated (“basal mutant”). All of the single-cysteine mutants in the sequence 107–135 were derivatives of this mutant, which are labeled as P107C–R135C/Basal mutant. As controls for the experiments, similar single-cysteine substituted mutants at amino acid positions 107–135 were generated on the background of wild type (WT) rhodopsin, containing the six natural cystein residues, which are labeled as P107C–R135C/WT.

The mutants P107C, T108C, G109C, and N111C were prepared by fragment replacement mutagenesis in the synthetic gene for bovine opsin [25] and cloned in the expression vector PMT4 [26]. The restriction fragments NcoI/XhoI (nucleotides 302–339) containing the single-cysteine substitutions of these constructs were used to replace the NcoI/XhoI fragment of the basic mutant. For mutants G114C, F115C, F116C, A117C, T118C, L119C, G120C, G121C, E122C, I123C, A124C, L125C, W126C, S127C, L128C, and V129C, DNA duplexes containing the cysteine codons (TGC) replaced the native restriction fragment XhoI/PvuI (nucleotides 339–403). For mutants V130C, L131C, A132C, I133C, E134C, and R135C, DNA duplexes containing the cysteine codons replaced the native restriction fragment speI/BsaAI (nucleotides 387–413).

PCR mutagenesis was used for preparation of the mutants L112C/Basal mutant and E113C/Basal mutant. The first step involved PCR reactions with the A132C/Basal plasmid as the template, using the following primers containing the above single-cysteine codon, one at a time: the primer 5′ CTG CAA GAA TTC ATG AAC GGT ACC GAA GGC CCA (EcoRI); the primer 5′ GAC TAC TAG TGA CCA CAG TGC AAT TTC ACC GCC CAG GGT GGC AAA GAA GCC CTC ***GCA*** GTT for L112C; and the primer 5′ GAC TAC TAG TGA CCA CAG TGC AAT TTC ACC GCC CAG GGT GGC AAA GAA GCC ***GCA*** GAG GTT (speI) for E113C (the cysteine-coding codon is in italics). The PCR products were digested to provide the EcoRI/speI fragments (nucleotides 2–387) containing the cysteine codons, which were then subcloned into the large fragment speI/EcoRI (nucleotides 388–6182) of the L119C/Basal mutant. The DNA sequences of the fragments containing the mutated regions in all constructs were confirmed by the dideoxynucleotide sequencing method.

### Expression and purification of rhodopsin

Transient transfection of COS-1 cells and treatment of the harvested cells with 11-*cis*-retinal were performed as previously described [24]. In brief, for expression of opsin in COS-1 cells, 15 µg of plasmid DNA was used to transfect a plate (150×25 cm) of confluent COS-1 cells and the cells were harvested 50–56 hours post-transfection and washed two times with buffer A. The cell pellets were stored at −78°C. For rhodopsin purification, the cell pellets were warmed up to 20°C and resuspended in buffer A containing 11-*cis*-retinal (final concentration: 25 µM) and 0.1 mM PMSF (2 mL/plate) at 4°C with end-over-end mixing for 4 h in the dark for generating the rhodopsin chromophore. Then the pellet cells were placed in the SORVALL RC-5 (8000 rpm, 4°C, 10 min) and solubilized in buffer B containing 1% DM and 0.1 mM PMSF for 1 h at 4°C. The suspension was centrifuged at 35,000 rpm for 30 min at 4°C in a Ti60 rotor to remove the insoluble material. Mutant rhodopsins were purified by using 1D4-sepharose 4B affinity chromatography as previously described [24]. The suspensions with beads were transferred to a disposable 2 mL polypropylene column with a frit. The beads were washed with 50 mL of buffer C, followed by 50 mL of buffer D. Rhodopsin was eluted with buffer E. The opsin expression and the purity of the purified rhodopsin were analyzed by SDS-PAGE and the expressed proteins were electrotransferred onto polyvinylidene fluoride transfer membrane and detected using the rho-1D4 monoclonal antibody using western blot analysis.

### Spectral analysis of WT and the mutant rhodopsins

The formation of the samples' chromophore was checked by the difference of UV-visible (UV/vis) absorption spectra before the rhodopsin was incubated with 1D4-antibody during the protein purification. UV-visible (UV-vis) absorption spectra of the purified proteins was recorded with a Perkin-Elmer λ-35 UV-vis spectrophotometer equipped with water-jacketed cuvette holders connected to a circulating water bath. All spectra were recorded with a bandwidth of 2 nm, a response time of 1 s, and a scan speed of 480 nm/min at 20°C either in the dark, after acidification, or after illuminating the samples for 30 sec with a fiber optic light equipped with a >495 nm long-pass filter. The molar extinction value (ε_500_) used for WT rhodopsin was 40,600 M^−1^cm^−1^ [22].

The rate of Meta II decay was measured by the fluorescence increase reflecting the retinal release [27]. Rhodopsin was pre-equilibrated in 200 µL of 2 mM Na-Pi (pH 6.0) and 0.05% DM at 20°C for 10 minutes, and its fluorescence increase was recorded after photobleaching for 30 sec. The excitation and emission wavelengths were 295 nm (slit = 0.25 nm) and 330 nm (slit = 12 nm), respectively.

### Reaction of rhodopsin-cysteine-mutants/Basal mutant with sulfhydryl specific reagent 4-PDS

The reaction of rhodopsin cysteine mutants with reagent 4-PDS is shown in Figure S1 [16]. Rhodopsin mutant samples and buffer F were mixed with 1 mM 4-PDS such that the final solution contained 0.5 µM rhodopsin and 25 µM 4-PDS. The reaction at 20°C was then followed by monitoring the absorption of the product 4-thiophridone at 323 nm in the dark and after illumination for 30 sec with the same concentration of 4-PDS in the reference cuvette. Each reaction was followed until completion, as indicated by no further increase of the absorption at 323 nm. The absorption of the rhodopsin alone at this wavelength was subtracted. The molar extinction coefficient of 4-thiopyridone at 323 nm was determined to be 19,000 M^−1^cm^−1^ by titration of L-cysteine with 4-PDS under the same conditions [23]. Due to the large excess of 4-PDS, the reaction is pseudo-first-order in sulfhydryl concentration. Therefore, for each reactive mutant, the increase in 4-thiopyridone as a function of time was fitted by a single-exponential function to determine the reaction rate constant. All experiments were independently performed at least in duplicate. The error given of the pseudo-first-order rate constant is the standard deviation.

### Vacuum electrostatics of Rhodopsin surface charge analysis

The 3-dimensional structure model and vacuum electrostatics analyses of rhodopsin were performed with Pymol and the structure of inactive and partially active rhodopsin [2], [7], [8].

## Results

### Spectral characterization of the cysteine mutants P107C-R135C/Basal mutant or WT

**Figure 2:**
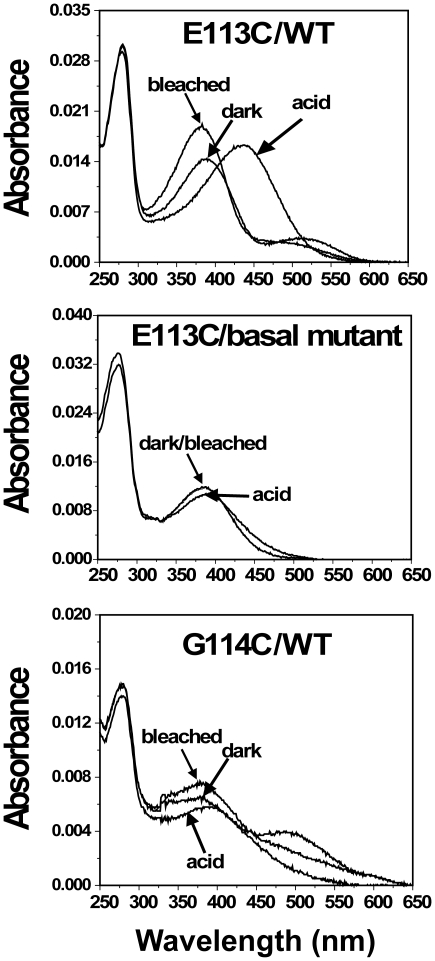
Analysis of UV-vis spectra of cysteine mutants E113C/WT, E113/Basal mutant, and G114C/WT. Mutants were purified from COS-1 cells after regeneration with 25 µM 11-cis-retinal. UV-vis spectra were recorded in the dark, after acidification, and after illuminating the samples for 30 sec at 20°C.

**Figure 3:**
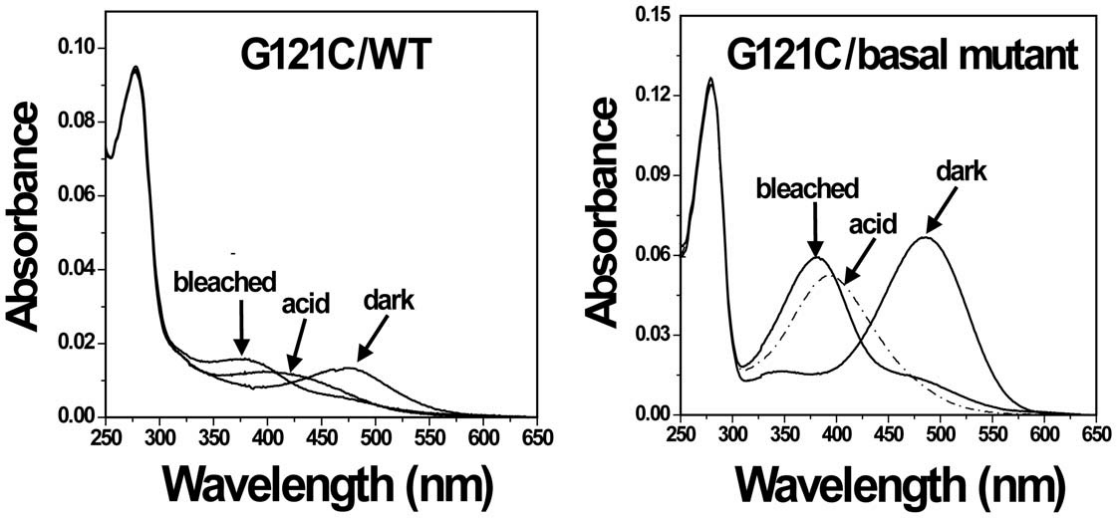
Analysis of UV-vis spectra of cysteine mutants G121C/WT and G121C/Basal mutant. Mutants were purified from COS-1 cells after regeneration with 25 µM 11-cis-retinal. UV-vis spectra were recorded in the dark, after acidification, and after illuminating the samples for 30 sec at 20°C.

As shown in Table 1, most of the purified-mutants/Basal mutant except for P107C/, N111C/, G114C/, A124C/, L125C/, W126C/, L128C/, and E134C/Basal mutant formed the typical rhodopsin-like chromophore with A_280_/A_500_ ratios between 1.7 and 2.5, with an absorption λ_max_ in the visible range varying from 480 nm to 496 nm. The A_280_/A_500_ ratio of mutants G109C/Basal mutant and R135C/Basal mutant was slightly higher, about 2.5. For mutant E113C/Basal mutant, the λ_max_ of its chromophore was 380 nm. Upon illumination, all mutants except for P107C/, N111C/, G114C/, A124C/, L125C/, W126C/, L128C/, and E134C/Basal mutant formed the characteristic Meta II intermediates. However, the bleaching behavior was incomplete after illuminating the samples for 30 sec, in contrast to the basal mutant. After acidification, most of the bleached samples formed the 440 nm absorbing protonated retinyl Schiff base, but the absorption λ_max_ of E113 C/Basal mutant and G121C/Basal mutant was changed to 390 nm (Figure 2 and 3). The purified rhodopsins were homogeneous as judged by 10% SDS-PAGE (Figure S2).

**Table 1:** Characterization of single cysteine substitution mutants P107C-R135C on the background of the basal mutant (C140A/167A/185A/222A264A/316A).

Mutants	Chromophoreλ_max_ (nm)	A_280_/A_500_ *	Meta II decay(t_1/2_, min)
WT	500	1.6	14.7
Basal mutant	495	1.7	41
P107C	-	-	-
T108C	495	1.9	16.3
G109C	495	2.4	16
N111C	-	-	-
L112C	495	1.8	15
E113C	380	2.0#	-
G114C	-	-	-
F115C	493	2.0	13
F116C	497	1.7	16
A117C	486	1.8	34
T118C	480	2.0	16.4
L119C	497	1.9	44.5
G120C	494	1.9	40
G121C	485	1.8	1.8
E122C	490	1.75	38
I123C	495	1.9	27
A124C	-	-	-
L125C	-	-	-
W126C	-	-	-
S127C	496	1.8	46
L128C	-	-	-
V129C	496	1.7	42
V130C	496	1.7	35
L131C	495	1.7	37
A132C	494	1.7	20
I133C	495	1.8	29
E134C	-	-	-
R135C	492	2.5	42
R135C	492	2.5	42

*The UV/Vis absorbance spectral ratios were determined after elution from the immunoaffinity cloumn at pH 6.0.

- The purified mutants P107C, N111C, G114C, A124C, L125C, W126C, L128C, and E134C did not form rhodopsin-like chromophore.

# the ratio of rhodopsin mutant E113C was 280∶380.

To examine whether the introduction of cysteine into the TM helix III affects Meta II decay of rhodopsin, the rate of retinal release that parallels the Meta II decay of the protein was analyzed by fluorescence spectroscopy. The rates of Meta II decay (t_1/2_) after illumination are shown in Table 1. For WT rhodopsin, the t_1/2_ was 14.7 min under the assay conditions (Figure S3), and the t_1/2_ for the cysteine mutants T108C/, G109C/, L112C/, F115C/,F116C/, and T118C/Basal mutant were similar to WT. The basal mutant showed significant increases in Meta II half-life (Figure S3), while the t_1/2_ for mutants A117C/, L119C/, G120C/, E122C/, S127C/, V129C/, V130C/, L131C/, and R135C/Basal mutant varied from 34 to 46 min, which is similar to that of the basal mutant (t_1/2_, 41 min) (Table 1). The Meta II half-life of rhodopsin mutants I123C/, A132C/, and I133C/Basal mutant were between WT and the basal mutant. The single-cysteine mutant G121C/Basal mutant (t_1/2_, 1.8 min) showed the fastest Meta II decay among rhodopsin mutants (Table 1).

The purified rhodopsin mutants P107C/, N111C/, G114C/, A124C/, L125C/, W126C/, L128C/, and E134C/Basal mutant did not form the rhodopsin-like chromophore. Immunoblotting analysis of the solubilized extracts of cells expressing each mutant showed that most of the mutants showed a similar level of opsin expression compared with that of the basal mutant (data not shown). The difference in UV-vis spectra confirmed that there was not the formation of chromophore in these cysteine mutants (Figure S4).

**Figure 4:**
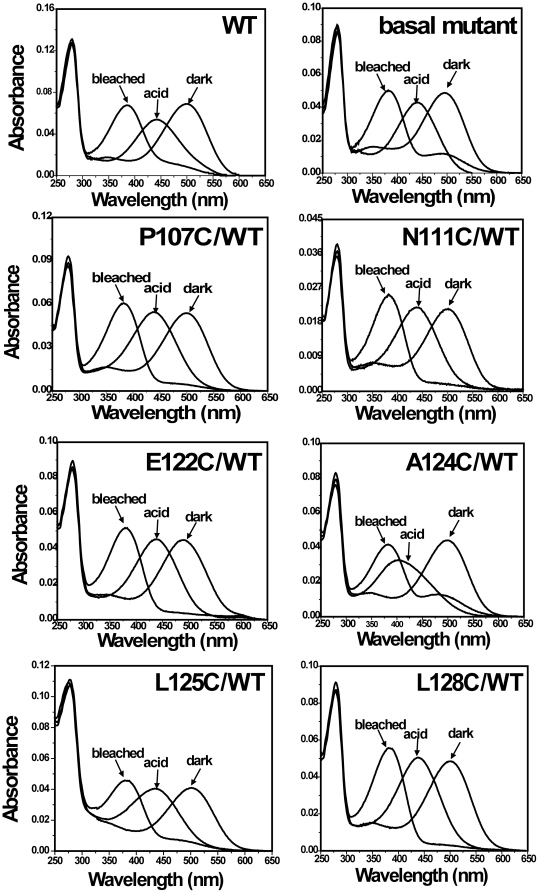
Analysis of UV-vis spectra of some selected single-cysteine mutants of rhodopsin on the background of wild-type rhodopsin. Mutants (Basal mutant, P107C/, N111C/, E122C/, A124C/, L125C/, and L128C/WT) were purified from COS-1 cells after regeneration with 25 µM 11-cis-retinal. UV-vis spectra were recorded in the dark, after acidification, and after illuminating the samples for 30 sec at 20°C.

**Figure 5:**
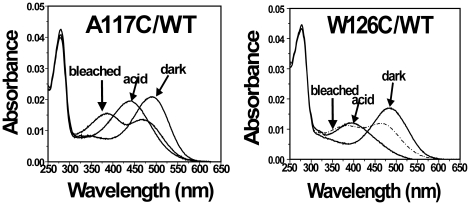
Analysis of UV-vis spectra of single-cysteine mutants A117C/WT and W126C/WT. Mutants were purified from COS-1 cells after regeneration with 25 µM 11-cis-retinal. UV-vis spectra were recorded in the dark, after acidification, and after illuminating the samples for 30 sec at 20°C.

Each of the single-cysteine mutants on the background of WT formed the typical rhodopsin-like chromophore except for G109C/WT; the formation of rhodopsin chromophore of single-cysteine-mutants/WT is representative (Figure 4). Upon illumination, all mutants/WT formed the characteristic Meta II intermediates. The rate of bleaching for all the mutants/WT was similar to WT except for the mutants A117C/WT and W126C/WT. Mutants A117C/WT and W126/WT formed the complex of Meta I and Meta II after illuminating the samples for 30 sec (Figure 5). Mutant E113C/WT regenerated with 11-cis-retinal and formed a pigment with λ_max_ = 380 nm with a small second component absorbing at 505 nm. But mutant G114C/WT only formed a pigment with λ_max_ = 380 (Figure 2). Upon illumination, the pigment was completely converted to the 380 nm form. Although single-cysteine mutant G121/WT formed the rhodopsin-like chromophore, the A_280_/A_500_ ratio was significantly higher at 6.4. Compared with cysteine mutant G121C/Basal mutant, the replacement of six natural cysteine residues by alanine led to an increase of A_280_/A_500_ ratios from 1.8 to 6.4 (Figure 3).

### Reactivity of the Cysteine sulfhydryl groups in mutants T108C-R135C/Basal mutant to 4-PDS

WT rhodopsin showed 2 mol of reactive cysteines per mole of rhodopsin (Cys140 and Cys316) in the dark [28]. The basal mutant (C140A/167A/185A/222A/264A/316A) incorporated ∼0.5 mole of the reagent per mole of rhodopsin in the dark or after illumination for 30 sec. The cysteine mutants L112C-A132C/Basal mutant incorporated approximately 0.5 mole of the reagent per mole of rhodopsin in the dark, which was similar to that of the basal mutant. The reaction was completed within several minutes, as little increase was detected upon further incubation. However, for mutants T108C/Basal mutant and G109C/Basal mutant, both incorporated ∼1.0 mole of 4-PDS per mole of rhodopsin, and mutants I133C/Basal mutant and R135C/Basal mutant incorporated ∼1.5 mole of the reagent per mole of rhodopsin in the dark.

**Figure 6:**
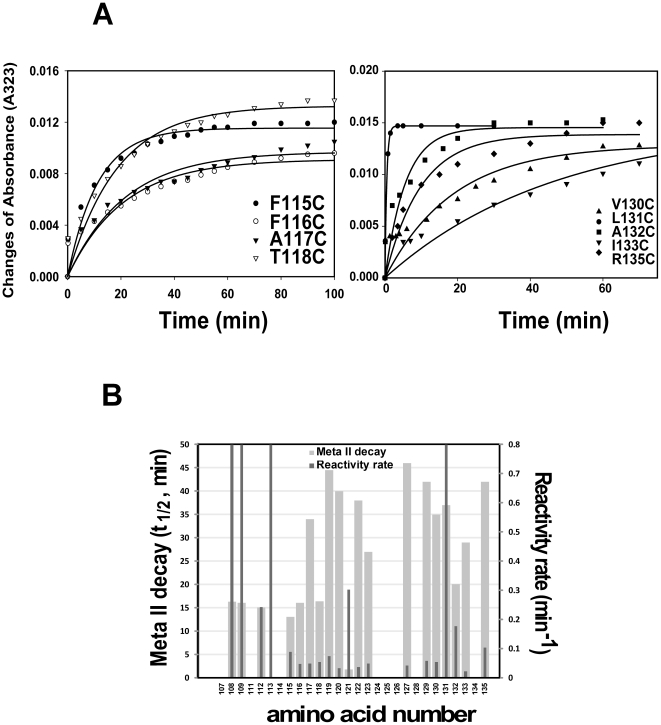
Comparison of PDS labeling rate among cysteine mutants and relationship analysis of PDS labeling rate with Meta II decay. **A**) Rates of cysteine reactivity with 4-PDS were evaluated in mutants (F115/, F116/, A117/, T118/, V130/, L131/, A132/, I133/, and R135/Basal mutant). The reaction was carried out with 0.5 µM of rhodopsin mutant and 25 µM 4-PDS in phosphate buffer (pH8.0) and 0.05% DM at 20°C. Time-dependent changes in absorbance at 323 nm after photoactivation were plotted. **B**) Rates of cysteine reactivity with 4-PDS (dark gray) and Meta II decay (light gray) in relationship to the amino acid position.

Upon photoactivation, these mutants incorporated ∼1 mole of the reagent per mole of rhodopsin over time after illuminating the samples for 30 sec. After light activation, comparison of the rate of reactivity calculated from absorbance increase at 323 nm indicated that mutants T108C/, G109C/, E113C/, and L131C/Basal mutant showed rapid reactions with 4-PDS. Reaction rate of mutants L112C/, G121C/, and A132C/Basal mutant with 4-PDS was moderate, while mutants F115C/, T118C/, L119C/, V129C/, V130C/, and R135C/Basal mutant showed a slow rate of reaction with 4-PDS. The slower reactions were observed with F116C/, A117C/, G120C/, E122C/, I123C/, S127C/, and I133C/Basal mutant. The cysteine mutant E113C/Basal mutant showed the fastest reaction rate with 4-PDS among all of the mutants at helix-III. The time courses for the reactions and exponential fits for selected mutants are shown in Figure 6A and Figure S5, and the pseudo-first-order rate constants for all of the mutants are listed in Table 2 and Figure 6B.

**Table 2:** Comparison of the reactivity of cysteine mutant T108C-R135C/Basal mutant at Helix-III of rhodopsin with 4-PDS after photoactivation.

mutant	Rate (min^−1^)^a^	mutant	Rate (min^−1^)^a^	mutant	Rate (min^−1^)^a^
T108C	>1.4^b^	T118C	0.054±0.006	V129C	0.057±0.006
G109C	>1.4^b^	L119C	0.074±0.008	V130C	0.053±0.011
L112C	0.242±0.0537	G120C	0.032±0.005	L131C	>1.4^b^
E113C	>1.4^b^	G121C	0.302±0.06	A132C	0.177±0.035
F115C	0.088±0.011	E122C	0.036±0.006	I133C	0.021±0.003
F116C	0.047±0.008	I123C	0.048±0.007	R135C	0.103±0.013
A117C	0.048±0.007	S127C	0.042±0.006	Basal mutant	0.041±0.005

a The error given of each value of the pseudo-first-order rate constant is the standard deviation.

b These reactions were complete at the first time point taken (∼0.5 min).

**Figure 7:**
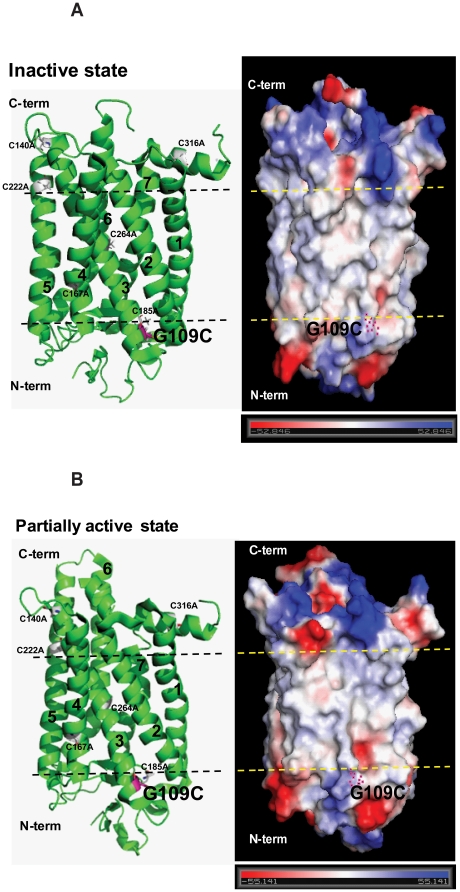
Vacuum electrostatics analysis of rhodopsin surface charge shows that hydrophobic/hydrophilic phase boundaries exist at position G109 of the intercellular domain. **A**) Left: cartoon and stick model of rhodopsin (inactive) with the basal mutants in gray and G109C in purple. Black lines show the hydrophobic/hydrophilic phase boundaries. Number 1–7 show the transmembrane helices. Right: vacuum electrostatics model of rhodopsin (inactive) shows the highly charged hydrophilic phase (negative in red and positive in blue) both in the cytoplasmic and intracellular areas and the low/non charged hydrophobic phase (between the yellow lines) in the membrane bilayer area. Purple-blue dots show the location of G109. **B**) Left: the cartoon and stick model of rhodopsin (partially active) with the basal mutants in gray and G109C in purple. Black lines show the hydrophobic/hydrophilic phase boundaries. Number 1–7 show the transmembrane helices. Right: vacuum electrostatics model of rhodopsin (partially active) shows the highly charged hydrophilic phase (negative in red and positive in blue) both in cytoplasmic and intracellular areas and the low/non charged hydrophobic phase (between the yellow lines) in the membrane bilayer area. Purple-blue dots show the location of G109. Bar (bottom) shows the negative and positive charge.

**Figure 8:**
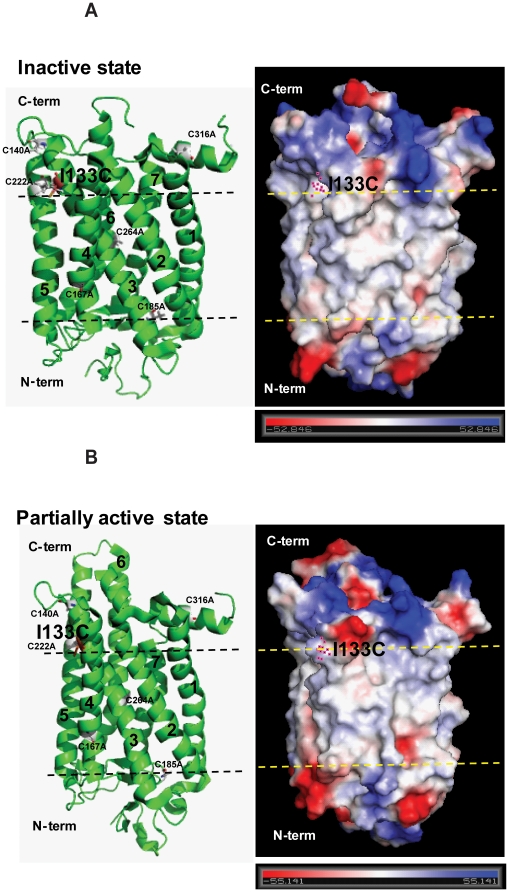
Vacuum electrostatics analysis of rhodopsin surface charge shows that hydrophobic/hydrophilic phase boundaries exist at positions of I133 at cytoplasm side. **A**) Left: the cartoon and stick model of rhodopsin (inactive) with the basal mutants in gray and I133C in brown. Black lines show the hydrophobic/hydrophilic phase boundaries. Number 1–7 show the transmembrane helices. Right: vacuum electrostatics model of rhodopsin (inactive) shows the highly charged hydrophilic phase (negative in red and positive in blue) both in cytoplasmic and intracellular areas and the low/non charged hydrophobic phase (between the yellow lines) in the membrane bilayer area. Purple-blue dots show the location of I133. **B**) Left: the cartoon and stick model of rhodopsin (partially active) with the basal mutants in gray and I133C in brown. Black lines show the hydrophobic/hydrophilic phase boundaries. Number 1–7 show the transmembrane helices. Right: vacuum electrostatics model of rhodopsin (partially active) shows the highly charged hydrophilic phase (negative in red and positive in blue) both in cytoplasmic and intracellular areas and the low/non charged hydrophobic phase (between the yellow lines) in the membrane bilayer area. Purple-blue dots show the location of I133. Bar (bottom) shows the negative and positive charge.

The big group including L112C-A132C/Basal mutant showed no reaction at all using prolonged times, but G109C/Basal and I133C showed completely reaction in the dark, which confirmed that hydrophobic/hydrophilic phase boundaries exist at positions of G109 and I133 of Helix-III. To determine whether the cysteine accessibility data agree with the accepted rhodopsin 3-dimensional structural model, two mutants GI09C/Basal mutant and I133C/Basal mutant were analyzed by vacuum electrostatics of rhodopsin surface charge changes in the inactive and partially active states (Figure 7 and Figure 8). The analysis data clearly demonstrated that hydrophobic/hydrophilic phase boundaries exist at positions of G109 and I133, which is consistent with the above-mentioned results. Furthermore, compared to the position changes at G109 and I133 between inactive and partially active structure, the helix-III showed little to no movement (Figure 7 and Figure 8).

## Discussion

The crystal structure of rhodopsin in the ground state provided the first detailed three-dimensional structural model for a GPCR [2], [29]. So far, only the crystal structure of partially activated rhodopsin has been resolved [7], [8]. Due to difficulties of getting the crystal structure of completely activated rhodopsin, several biochemical approaches have been employed to analyze its structural changes upon activation. In particular, cysteine scanning mutagenesis followed by analysis of reactivity of cysteine sulfhydryl groups has been extremely useful for delineating structural features of rhodopsin. The accessibility and reactivity measurement of single cysteines, introduced into various positions of the molecule, provides useful clues not only for the secondary and tertiary structural features of rhodopsin in the dark and upon photoactivation but also for the aqueous/TM boundary of the protein molecule.

Light-catalyzed retinal isomerization causes specific movements in the TM helices, resulting in a conformational change in the CP domain preparing the molecule for the protein-protein interactions involved in signal transduction [30], [31]. Rhodopsin is composed of a seven-TM helical protein domain (Figure 1). TM Helix-III is of significance from several points of view: 1) The larger conformational changes occur at the interhelical loops near the ends of the TM helices III and VI [31], which contribute to the binding site of the C-terminus of transducin α; 2) Helix-III provides many of the amino acid side chains that form the chromophore-binding pocket: E113, G114, A117, T118, G120, and G122 [32], and G121 is an important and specific component of the 11-cis-retinal binding pocket in rhodopsin [33], [34]; E113 serves as 11-cis-retinal Schiff base counterion [35]; 3) Cys-110 and Cys-187 form a disulfide bond to stabilize the rhodopsin structure; 4) The tripeptide E134-R135-Y136 is part of a highly conserved (D/E)R(Y/W) motif found in GPCRs, which participates in several hydrogen bonds with surrounding residues [2], [7], [8]. Proton uptake involves the ERY motif near the cytoplasmic surface of TM Helix-III during formation of Meta II [11], [36]. The rhodopsin activation in membranes is regulated by two protonation switches, including disruption of an interhelical salt bridge by internal proton transfer from the retinal protonated Schiff base to its counterion E134, and uptake of a proton from the solvent by E134 [10]. The conserved NpxxY(x)_5,6_F (D/E)R(Y/W) motif provides structural constraints in rhodopsin that rearrange in response to photoisomerization during formation of the G protein-activating Meta II [37].

As shown in Figure 4, the basal mutant regenerated with 11-cis-retinal similarly to the WT, with an A_280_/A_500_ ratio of 1.7 and an absorption visible maximum slightly blue-shifted to 495 nm. Thus, 11-cis-retinal binding to the basal mutant is near normal, the structure of the basal mutant is similar to that of WT rhodopsin and the substituted alanines lie in a similar orientation to that of the WT cysteine residues [38]. However, the basal mutant markedly differed from WT in Meta II half-life showing significant increase (∼41 min) (Figure S3). Namely, the rate of retinal release of the basal mutant is slower than that of WT. The alanine-substitution mutant prolonged the Meta II decay, which from an experimental point of view is very convenient to determine reactivity of the cysteine sulfhydryl groups in single-cysteine mutants on the background of the basal mutant to 4-PDS after photoactivation.

Two of the studied mutants, G109C/Basal mutant and R135C/Basal mutant, regenerated with 11-cis-retinal to different levels when compared to the WT (Table 1). The A_280_/A_500_ ratio was significantly increased to ∼2.5, indicating some degree of misfolding for these two mutants. This concurs with the previously reported spectrum of R135L [39] and is different from other reported mutations at amino acid position R135, such as R135G, R135T [40], or R135Q [35], respectively. This differential effect suggests that the size chain at position 135 may play a crucial role in rhodopsin folding. In addition, R135 formed a salt-bridge with E134 and E247 at the top of TM helices III and VI, holding these two helices together at their cytoplasmic ends and maintaining the receptor in its inactive state [2], [39]. Thus, the R135C/Basal mutant mutation would be disrupting this ionic interaction, resulting in partially misfolding and a reduced ability of the mutant protein to bind 11-cis-retinal. In the case of mutant G109C/Basal mutant, the inserted amino acid is close to a conserved disulfide bond (Cys110–Cys187) in rhodopsin. The higher A_280_/A_500_ ratio indicates that a cysteine substitution at position 109 may affect the correction matching of the disulfide bond. In the control experiment, G109C/WT did not form the rhodopsin-like chromophore, but G109C/Basal mutant formed the chromophore. This result suggests that the amino acid cysteine at position 109 may form an erroneous disulfide bond with cysteine at either position 185 or 187 and results in the misfolding of the protein.

The rate of Meta II decay of mutant G121C/Basal mutant (1.8 min) was the fastest among the cysteine mutants (Table 1). Acidification resulted in reprotonation of the Schiff base linkage at the visible band shifted to 390 nm. In addition, compared with single-cysteine mutant G121C/WT, we note that the G121C/Basal mutant led to an increase of A_280_/A_500_ ratios from 1.8 to 6.4 (Figure 3). G121 is close to F261 (helix-VI) in the rhodopsin crystal structure; they pair to form one boundary of the retinal-binding site, defining the C_4_-C_5_-C_18_ orientation (F261 C_z_-G121C_α_ distance, 5 Å; F261 C_z_-retinal C_4_ distance, 3.7 Å) [2]. This portion of the retinal-binding pocket around helices III and VI appears to be held rigidly together by tight van der Waals interactions [32]. The interaction between G121 and retinal is consistent with mutagenesis experiments in which replacement of G121 by cysteine caused blue-shifted λ_max_ values (500 nm→485 nm) and decreased retinal binding that corresponded to the bulk of the substituted side chain. G121 also is a specific component of the 11-cis-retinal binding pocket in rhodopsin [33]. These data can explain why the mutant G121C/Basal mutant contains a faster rate of Meta II decay and why it is different from UV-vis spectrum. Evidence also showed that second-site replacement of F261 by alanine caused a reversion of the loss-of-function G121 mutant phenotypes, which is consistent with the interpretation that G121 and F261 may interact to form part of the retinal-binding pocket [34]. The six natural cysteine (C140→C-II, C167→Helix III, C185→E-II, C222→Helix V, C264→Helix VI, and C316→Helix VIII) replacements by alanine resulted in a recovery of the loss of function of single-cysteine mutant G121C/WT (Figure 3). Further studies will be performed to clarify which cysteine is the key amino acid to recover the function of this mutant.

In the current report, the substitution of E113 by cysteine (E113C/WT) also formed a markedly blue-shifted pigment (λ_max_ = 380 nm) and a slightly red-shifted pigment (λ_max_ = 505 nm) (Figure 2). The pigment (λ_max_ = 505 nm) of the mutant E113C/Basal mutant completely converted to λ_max_ = 380 nm (Figure 2). This is similar to mutant E113Q forming two pigments that were composed of λ_max_ = 380 nm and λ_max_ = 490 nm. The 380-nm species of mutant E113Q existed in a pH-dependent equilibrium with a 490-nm species such that at acidic pH all of the pigment was converted to λ_max_ = 490 nm. However, for mutant E113C/WT subsequent acidification resulted in reprotonation of the Schiff base linkage as the visible band shifted back to 440 nm (Figure 2). Mutant E113C/Basal mutant only formed a pigment (λ_max_ = 380 nm). After acidification, the chromophore changed to 390 nm (Figure 2). The E113 is unprotonated and negatively charged in the ground state of rhodopsin [41]. However, E113 becomes protonated upon light-dependent formation of Meta II and is the net proton acceptor for the Schiff base proton [42]. The pI of the amino acids cysteine and glutamine is 5.15 and 5.65, respectively, higher than that of glutamic acid (pI = 3.08). Thus, the substitution of glutamic acid-113 by glutamine and cysteine showed similar UV-vis spectra, but having a different spectra characterization as compared with WT.

The mutant G114C/WT formed similar UV-vis spectra to E113C/Basal mutant, showing a pigment in light absorption (λ_max_ = 380 nm) (Figure 2). The amount of protein recovered from the immunoaffinity column is very low when compared with that of WT. The lower A_280_/A_500_ ratio indicates the misfolding of G114C/WT. In addition, the mutants at position 114 have been shown not to regenerate with 11-cis-retinal [33], [39]. Because G114 is a key amino acid to form retinal binding pocket, the substitution of glycine-114 by cysteine reduced the binding ability of rhodopsin to 11-cis-retinal.

Mutants A117C/WT and W126C/WT formed the complex of Meta I and Meta II after illuminating the samples for 30 sec (Figure 5). The chromophore of W126C/WT red-shifted to λ_max_ = 484 nm. Increasing illumination time resulted in more Meta II formation. UV-absorption spectroscopy has suggested that the local protein environment around Trp residues changes during the conversion of rhodopsin to Meta II, and a linear dichroism study of UV-difference bands indicated a reorientation of an indole side chain of Trp during the Meta I to Meta II conversion [32].

Mutants P107C, N111C, G114C, A124C, L125C, W126C, L128C, and E134C on the background of the basal mutant failed to regenerate with 11-cis-retinal. The absence of chromophore regeneration for these mutants was confirmed by western blotting and UV-vis absorption spectra (Figure S4). However, the above-mentioned single cysteine mutants/WT can form the chromophore (Figure 4).

A number of factors could affect the reactivity of the sulfhydryl groups in different cysteines, including solvent accessibility, disposition within the structure, and the dielectric constant prevailing in the immediate environment [23]. In the dark, one big group, which showed no reaction at all using prolonged times, is composed of L112C-A132C/Basal mutant except for E113C/Basal mutant. Lack of reactivity of a sulfhydryl group in a cysteine indicates that the residue is buried either in a tertiary structure or in the hydrophobic micelle interior. According to our accessibility data, an aqueous/hydrophobic boundary in helix-III is near G109 and I133. Vacuum electrostatics analysis of rhodopsin surface charge clearly showed that hydrophobic/hydrophilic phase boundaries exist at positions of G109 and I133 (Figure 7 and Figure 8). Furthermore, the second proton at E134 during formation of Meta II has been shown to be functional in the isolated synthetic Helix-III and operates specifically at the phase boundary [11], which is of direct relevance to the placement of the phase boundary at I133 in cysteine accessibility data.

Another group, which showed markedly different reaction rates with 4-PDS, includes T108C/, G109C/, E113C/, and L131C/Basal mutant. The high accessibility of cysteine in mutant E113C/Basal mutant with 4-PDS in the dark demonstrated that the internal salt bridge with the Schiff base was broken by a cysteine substitution at E113, which opened the opsin structure already in the dark. The rates of reaction with 4-PDS upon illumination are shown in Table 2 for all of the cysteine mutants, and kinetic plots for selected mutants are showed in Figure 6A and supplementary Figure 5. Wide variation in reactivity was observed among cysteine mutants at different positions in the sequence 108–135 after photoactivation. Mutants E113C/, T108C/, G109C/, and L131C/Basal mutant reacted so rapidly that their reactions were complete at the first time point taken (∼0.5 min). A second group, comprising L112C/, G121C/, and A132C/Basal mutant, showed variations, but the rates were intermediate. Cysteines at positions 116, 117, 120, 122, 123, 127, and 133 reacted extremely slowly with 4-PDS, and mutants F115C/, T118C/, L119C/, V129C/, V130C/, and R135C/Basal mutant reacted with 4-PDS faster than those of F116C/Basal mutant. For mutants L112C-A132C/Basal mutant, excepting E113C/Basal mutant, the lack of reactivity in the dark and the accessibility of cysteine after photoactivation indicate the increases of water/4-PDS accessibility for certain cys-mutants. Comparing the position changes at G109 and I133 between inactive and partially active structure by vacuum electrostatics analysis of rhodopsin surface charge, the helix-III showed little movement (Figure 7 and Figure 8), which is consistent with a pattern of helix switch due to activation [9], [13]; namely, the activated rhodopsin has an outward movement of TM-VI, and smaller movements involving TM-I, TM-V, TM-VII, and TM-VIII, while the other helices remain largely unchanged.

The relationship between the mutant Meta II decay and the cysteine accessibility in these mutants/Basal was also addressed (Figure 6B). The mutant Meta II decay was mainly dependent on their location at Helix-III. Most of the mutants affecting amino acids close to TM N-terminus, including T108C, G109C, L112C, F115C, F116C, and T118C/Basal mutant showed a short Meta II decay (t_1/2_∼16 min), which is similar to that of WT (t_1/2_, 14.7 min), whereas most of mutants involving amino acids near the TM C-terminus, including L119C-R135C/Basal mutant, showed a long Meta II decay (t_1/2_∼40 min), which is similar to that of basal mutant (t_1/2_, 41 min) (Table 1 and Figure 6B). However, according to the accessibility data of the buried residues in the membrane bilayer, most of mutants showed a slow reaction rate with 4-PDS within membrane bilayer (Figure 6B and Table 2). Thus, it does not seem to exist a relationship between the Meta II decay of the mutants and the cysteine accessibility.

Water reorganization following photoactivation releases the constraints mediated by ionic lock (ERY motif), and a secondary internal hydrogen bonding network (Y206-H211-M163-E122), which may imply movement of water molecules and/or side chains and structural alterations of the helices [12]. The distribution and functional plasticity of water molecules in the TM region contribute to the absence of large conformational changes in rhodopsin after photoactivation [13], [14]. Photoactivation disrupts and reorganizes multiple constrains mediated by side chains and bound water, which transmits signaling from the chromophore and opens a groove on the cytoplasmic surface of the receptor, finally promoting catalytic exchange of GDP to GTP in transducin α. An increase of water/4-PDS accessibility for certain cys-mutants at Helix-III during formation of Meta II indicates an opening along the region of binding 11-cis-retinal. According to Altenbach's and Grossfield's recently published studies [9], [14], it is possible to predict that the minimal “opening” of the heptahelical structure for 4-PDS accessibility on water uptake is approximately 3–5 Å after light activation.

In summary, our data show that an aqueous/hydrophobic boundary in helix-III is near G109 and I133. The chromophore-facing residues of Helix-III become water-accessible after photoactivation.

## Supporting Information

Figure S1 Schematic representation of the rhodopsin cysteine mutants with 4-PDS. The reaction product thiopyridone is maximum at 323 nm. Thus, the reaction kinetics and the number of reactive cysteines can be determined by monitoring the absorption at this wavelength.
(TIF)

Figure S2 10% SDS-PAGE analysis of the purified cysteine mutants on the background of basal mutant.
(TIF)

Figure S3 Meta II decay of wild type rhodopsin and basal mutant. The changes in fluorescence were measured in a buffer containing 2 mM Na-Pi (pH 6.0) and 0.05% DM after illuminating the samples for 30 sec at 20°C.
(TIF)

Figure S4 UV-Vis spectra of rhodopsin cysteine mutant L125C/Basal mutant and E134C/Basal mutant. Mutants were purified from COS cells after regeneration with 25 µM 11-cis-retinal. UV-Vis spectra were recorded in the dark with rhodopsin eluted in a buffer containing 2 mM NaPi (pH6.0), 0.05%DM, 100 µM C' 1–9 peptide and 100 mM NaCl.
(TIF)

Figure S5 Comparison of PDS labeling rate among cysteine mutants. These mutants include T108/, G109/, L112/, E113/, L119/, G120/, G121/, E122/, and I123/Basal mutant. The reaction was carried out with 0.5 µM of rhodopsin mutant and 25 µM 4-PDS in phosphate buffer (pH8.0) and 0.05% DM at 20°C. Time-dependent changes in absorbance at 323 nm after photoactivation were plotted.
(TIF)
