# Supplementary figures and images for: The Roles of Transmembrane Domain Helix-III during Rhodopsin Photoactivation
Source: PLoS One. 2011 Feb 25;6(2):e17398. doi: 10.1371/journal.pone.0017398 (PMC3045455; doi:10.1371/journal.pone.0017398)

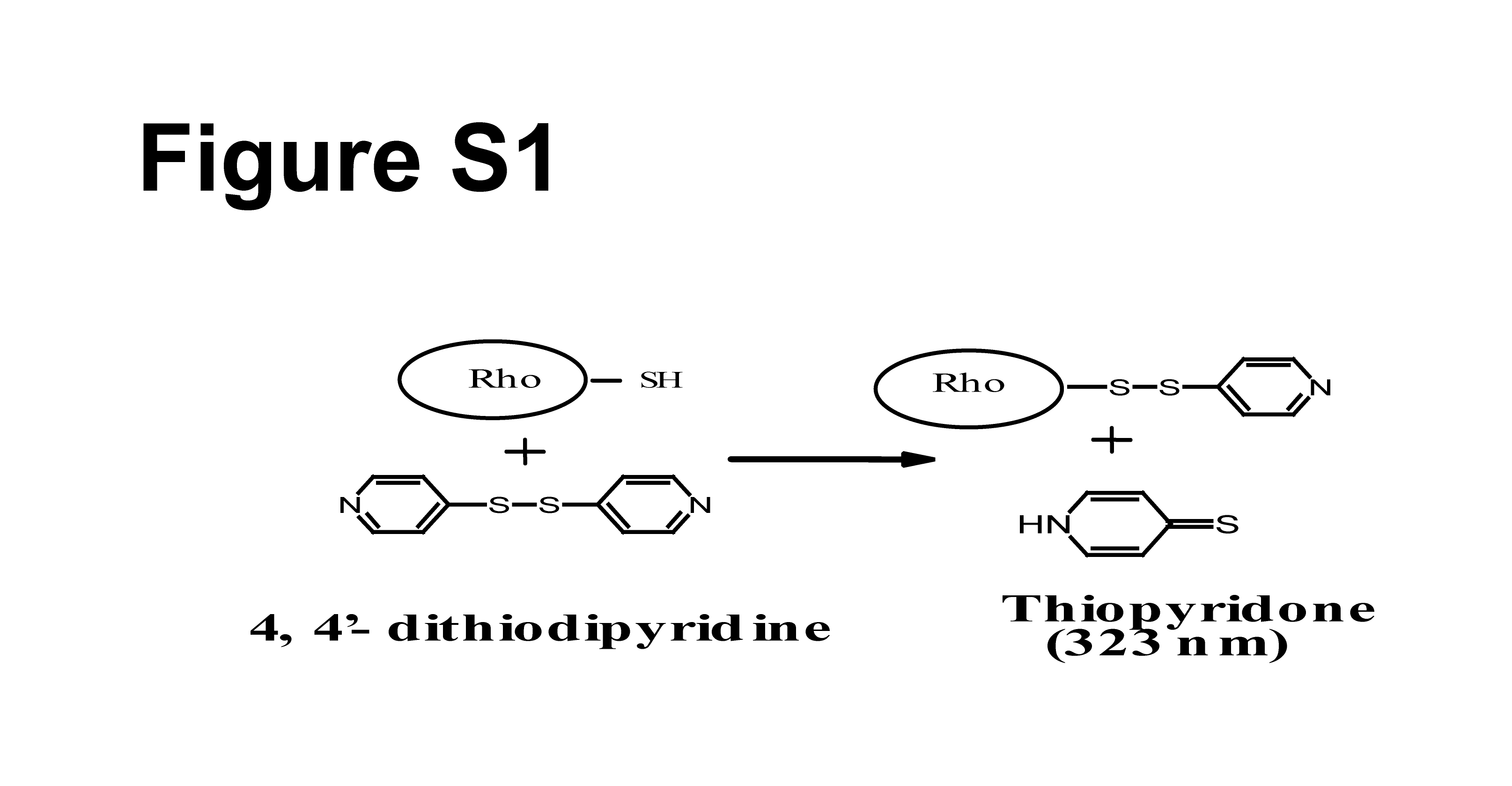

Supplement: Figure S1 — Schematic representation of the rhodopsin cysteine mutants with 4-PDS. The reaction product thiopyridone is maximum at 323 nm. Thus, the reaction kinetics and the number of reactive cysteines can be determined by monitoring the absorption at this wavelength. (TIF) [file pone.0017398.s001.tif]

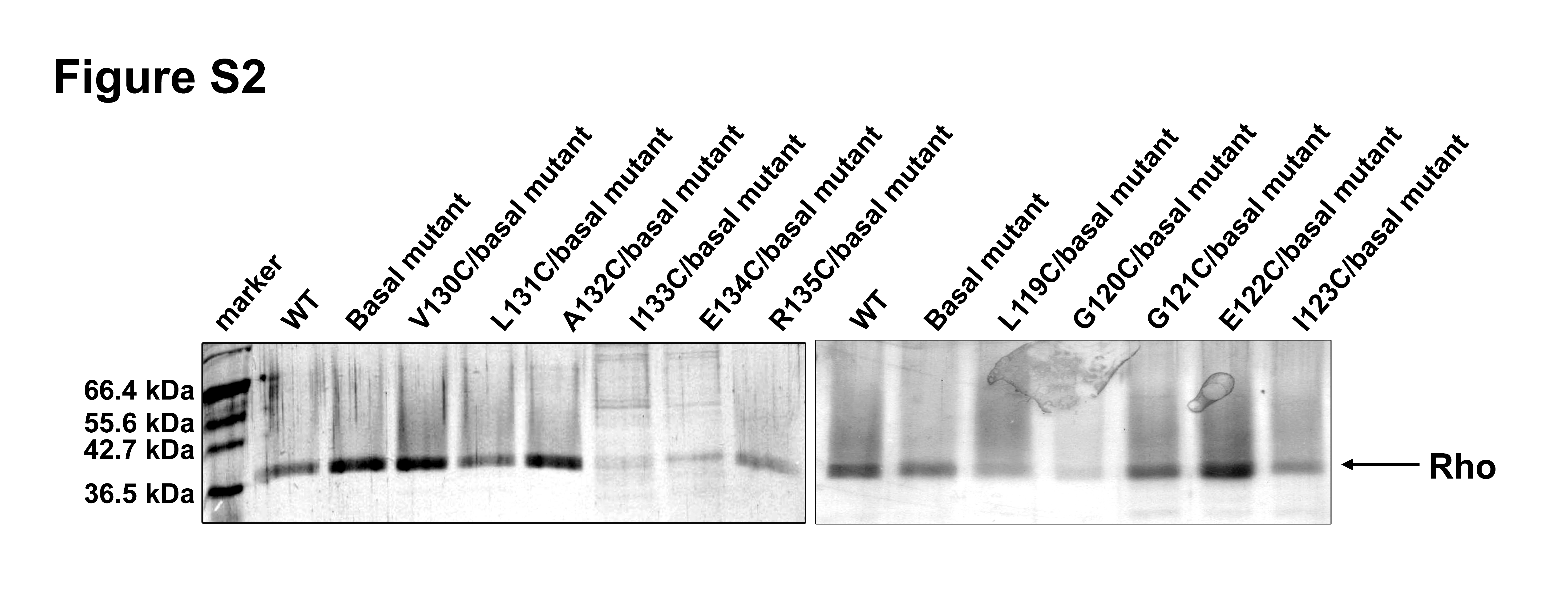

Supplement: Figure S2 — 10% SDS-PAGE analysis of the purified cysteine mutants on the background of basal mutant. (TIF) [file pone.0017398.s002.tif]

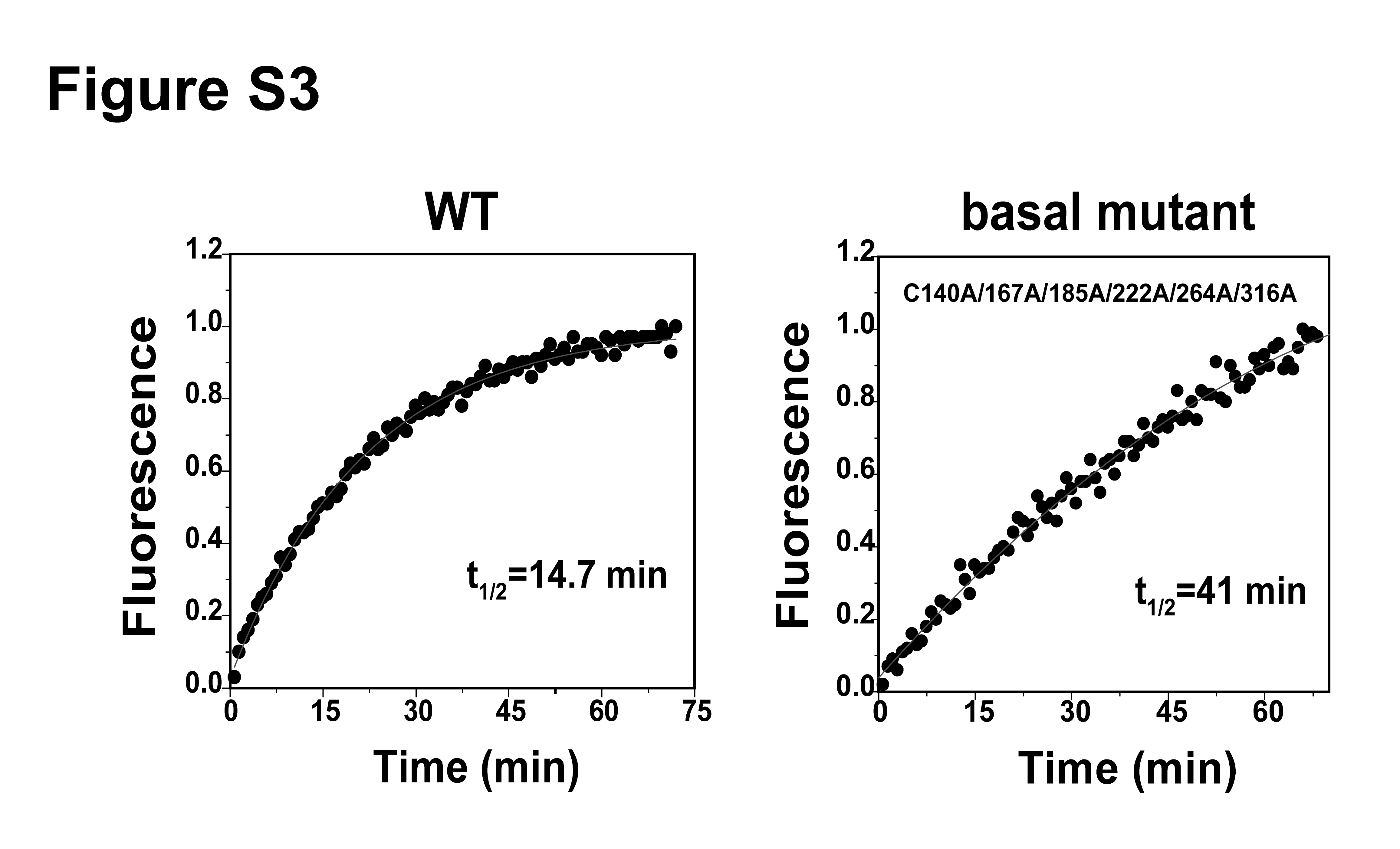

Supplement: Figure S3 — Meta II decay of wild type rhodopsin and basal mutant. The changes in fluorescence were measured in a buffer containing 2 mM Na-Pi (pH 6.0) and 0.05% DM after illuminating the samples for 30 sec at 20°C. (TIF) [file pone.0017398.s003.tif]

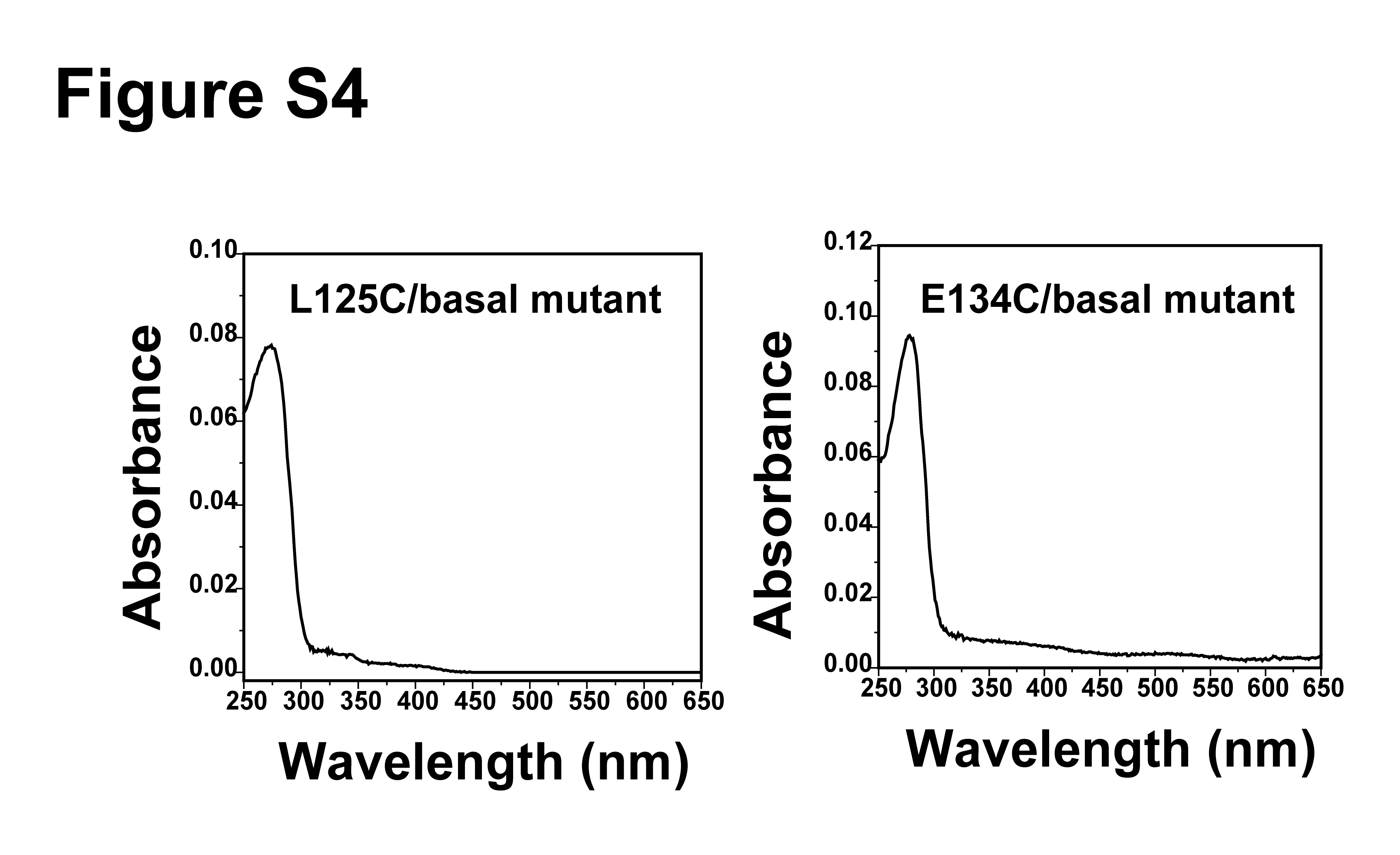

Supplement: Figure S4 — UV-Vis spectra of rhodopsin cysteine mutant L125C/Basal mutant and E134C/Basal mutant. Mutants were purified from COS cells after regeneration with 25 µM 11-cis-retinal. UV-Vis spectra were recorded in the dark with rhodopsin eluted in a buffer containing 2 mM NaPi (pH6.0), 0.05%DM, 100 µM C' 1–9 peptide and 100 mM NaCl. (TIF) [file pone.0017398.s004.tif]

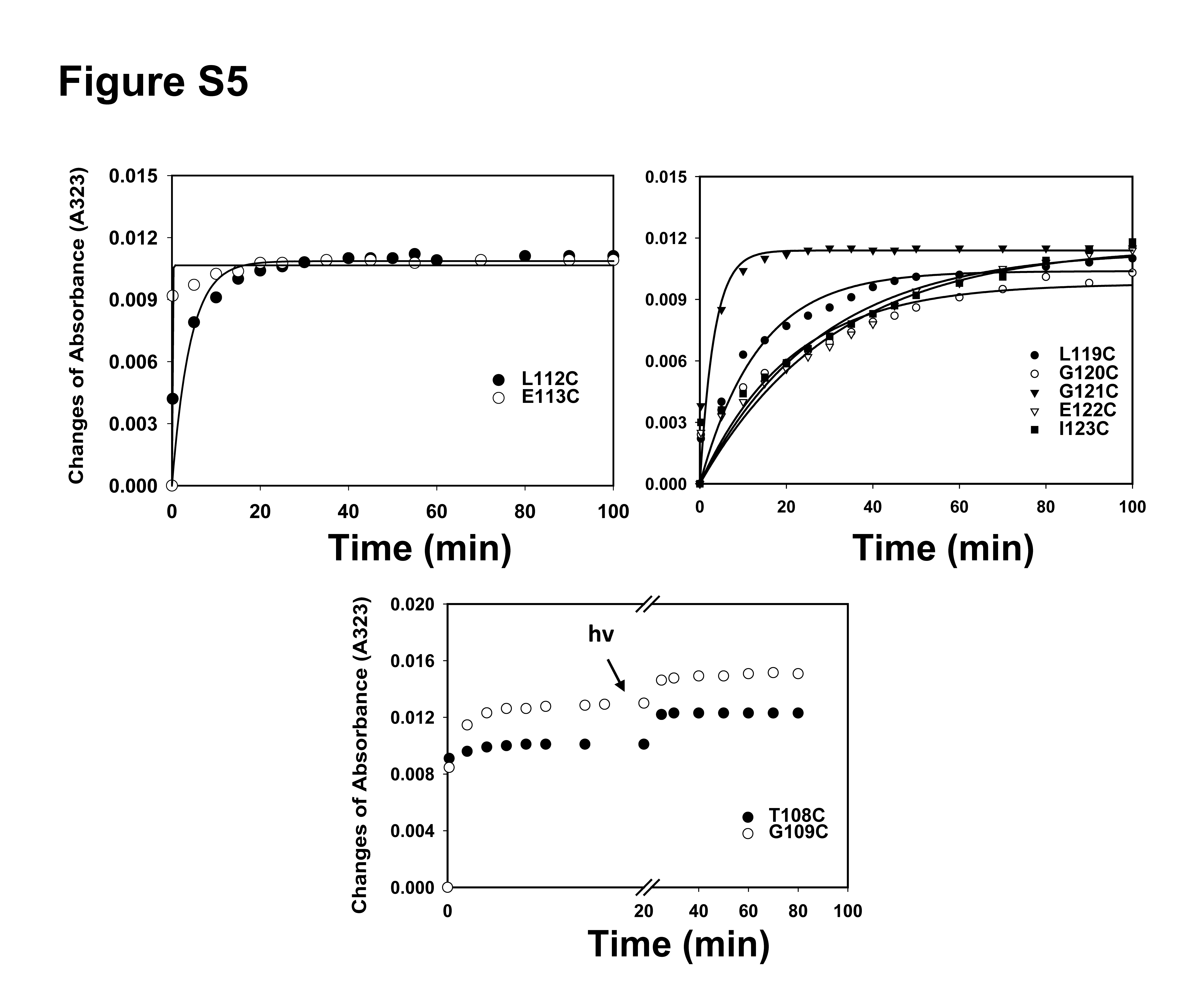

Supplement: Figure S5 — Comparison of PDS labeling rate among cysteine mutants. These mutants include T108/, G109/, L112/, E113/, L119/, G120/, G121/, E122/, and I123/Basal mutant. The reaction was carried out with 0.5 µM of rhodopsin mutant and 25 µM 4-PDS in phosphate buffer (pH8.0) and 0.05% DM at 20°C. Time-dependent changes in absorbance at 323 nm after photoactivation were plotted. (TIF) [file pone.0017398.s005.tif]
